# Breast Implant-Associated Immunological Disorders

**DOI:** 10.1155/2022/8536149

**Published:** 2022-05-04

**Authors:** Lily J. Suh, Imran Khan, Christine Kelley-Patteson, Ganesh Mohan, Aladdin H. Hassanein, Mithun Sinha

**Affiliations:** ^1^Department of Surgery, Indiana Center for Regenerative Medicine and Engineering, Indiana University School of Medicine, Indianapolis, IN 46202, USA; ^2^Meridian Plastic Surgeons, Indianapolis, IN 46290, USA

## Abstract

**Background:**

Breast implants are commonly placed postbreast cancer reconstruction, cosmetic augmentation, and gender-affirming surgery. Breast implant illness (BII) is a systemic complication associated with breast implants. Patients with BII may experience autoimmune symptoms including fatigue, difficulty concentrating, hair loss, weight change, and depression. BII is poorly understood, and the etiology is unknown. The purpose of this literature review is to characterize BII autoimmune disorders and determine possible causes for its etiology.

**Methods:**

The PubMed, Google Scholar, Embase, Web of Science, and OVID databases were interrogated from 2010 to 2020 using a query strategy including search term combinations of “implants,” “breast implant illness,” “autoimmune,” and “systemic illness.”

**Results:**

BII includes a spectrum of autoimmune symptoms such as fatigue, myalgias/arthralgias, dry eyes/mouth, and rash. A review of epidemiological studies in the past ten years exhibited evidence affirming an association between breast implants and autoimmune diseases. The most commonly recognized were Sjogren's syndrome, rheumatoid arthritis, systemic sclerosis, chronic fatigue syndrome, and Raynaud's syndrome. Explantation resulted in alleviation of symptoms in over 50% of patients, strengthening the hypothesis linking breast implants to BII. Studies have shown that silicone is a biologically inert material and unlikely to be the cause of these symptoms. This is supported by the fact that increased risk of autoimmune disease was also reported in patients with other implantable biomaterials such as orthopedic implants. Recent studies shed light on a possible role of bacterial biofilm and subsequent host-pathogen interactions as a confounding factor to this problem.

**Conclusion:**

BII could be dependent on biofilm infection and the microenvironment around the implants. The true pathophysiology behind these complaints must be further investigated so that alternative treatment regimens other than explantation can be developed. Translational significance of these studies is not limited to breast implants but extends to other implants as well.

## 1. Introduction

### 1.1. History

**Figure 1:**
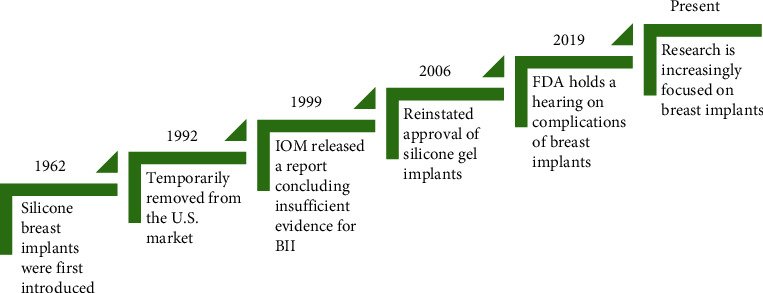
History of breast implant research.

Breast implants are frequently placed postbreast cancer reconstruction, cosmetic augmentation, and gender-affirming surgery. Breast implant illness (BII) is a term adopted by patients and physicians to refer to the range of systemic symptoms that occur after breast implant placement in lieu of an official diagnosis for these illnesses. These symptoms commonly included fatigue, fever, myalgias, and arthralgias [1, 2]. After silicone breast implants were first introduced in 1962, concerns over the reports of BII-type symptoms experienced by some patients with breast implants led to the Food and Drug Administration (FDA) to remove silicone implants from the US market in the 1990s (Figure 1). However, the Institute of Medicine (IOM) released a report in 1999 concluding there is insufficient evidence to support an association of silicone breast implants with connective tissue or rheumatic disease or a novel syndrome [3, 4]. In 2006, the US Food and Drug Administration reinstated the approval of silicone gel implants in breast reconstruction [4].

However, the reports of these systemic symptoms continue to arise. Given that the symptoms begin after placement of the implant and are sometimes relieved by explantation, some patients and physicians have attributed the nonspecific symptoms to the breast implants [5, 6]. Other terms used to describe this phenomenon include human adjuvant disease, adjuvant breast disease, or autoimmune/inflammatory syndrome induced by adjuvants (ASIA) [7–9]. In 2019, women affected by breast implant complications such as breast implant-associated anaplastic large cell lymphoma (BIA-ALCL) spoke at a national FDA hearing on their negative experiences with breast implants [10], which sparked greater impetus for increased research on the complications of breast implants. In 2020, the FDA reported that they received 2,497 medical device reports of breast implants causing symptoms consistent with BII between November 2018 and October 2019 [11], illustrating the growing scope of this problem.

### 1.2. Controversy

The data implicating breast implants and BII has been limited to potential associations. No mechanism or etiology has been shown. Thus, it has become a source of debate whether breast implant illness is a legitimate medical diagnosis. Articles debating the reality of breast implant illness are continually imbued with “Is Breast Implant Illness a Myth?” and other comparable titles [12, 13]. Although studies have been done that show evidence for increased risk of negative symptoms in women with breast implants [1, 14, 15], there have also been a number of studies that are skeptical if this increase in risk is statistically significant. Literature reviews of studies have also criticized study design of research articles that report positive risk for BII symptoms [16]. Questionnaires that have patients self-report their symptoms are utilized in many research studies to determine risk of symptoms associated with breast implants, but it has been argued that self-reported symptoms are prone to selection bias [17].

Due to the fluctuating public perspectives on the legitimacy of this phenomenon, women who identify as BII patients express feelings of invalidation and dismissal by physicians [6], especially as a subset may be referred for psychiatric evaluation [18]. Women who have had breast implant complications have expressed concerns and frustrations with their breast implant procedures on social media platforms [19]. With increased research into breast implant illness, there has been mounting evidence for an association between autoimmune symptoms and breast implants [15, 17, 20–24]. This is illustrated and discussed in detail in this review. It is imperative to have more large-scale epidemiological studies that will further settle the controversy. In October 2021, before the US Plastic Surgery meeting (PSTM 2021, Atlanta), FDA issued box label guidelines for implants to alert the patients of possible BIA-ALCL and BII prior to the placement of implants through a written and signed consent both by the patient and surgeon [25].

### 1.3. Significance

Nearly 300,000 women have breast implant surgeries every year in the United States, either for reconstruction or cosmetic purposes [26]. The number of patients who opt for breast implant explantation due to complications including BII is over 30,000 and increasing every year [26]. However, explantation is not an ideal treatment for patients may be left with a chest deformity if they have had a mastectomy or significant breast ptosis if for augmentation. Women have breast implants placed for reasons such as cosmetic augmentation, gender affirmation, or reconstructive surgery after breast cancer treatment or prophylactic mastectomy. Evaluations of women after implant placement have shown that breast implants, particularly silicone breast implants, may improve quality of life and body image in recipients [27–29]. Proper understanding of the etiology of these complaints is needed so that effective treatment regimens other than explantation can be developed.

It is also possible that “breast implant illness” is not in fact a phenomenon that is not limited to breast implants, but also implants of different biomaterials [30]. Implantation of different materials are utilized in a plethora of medical reconstructive procedures [31, 32]. Substances such as orthopedic implants have been associated with systemic symptoms and autoimmune disorders similar to those found in patients with breast implants [33–35]. This sparks the question: Could the autoimmune response seen in bioimplants actually be independent of the implant material and more dependent on microenvironment around the implant? Answering this question and understanding the pathophysiology of breast implant illness may have translational significance that extends to implants of other materials as well.

## 2. Methods

**Figure 2:**
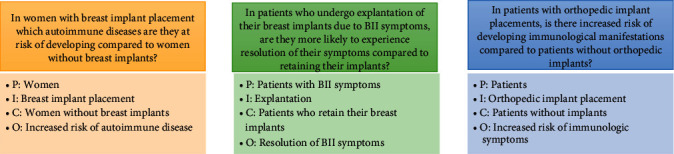
Depiction of questions investigated in present literature review.

We performed a literature review with the aim of characterizing the relationship between breast implants and autoimmune disease or symptoms, to review relevant literature to determine whether there is a consensus to which autoimmune diseases are associated with breast implants. We also looked at the efficacy of breast implant explantation as a treatment for breast implant illness. Lastly, we investigated the existence of similar symptoms as breast implant illness in other medical devices such as orthopedic or other implants. Further discussion is then provided on possible hypotheses for the etiology of breast implant illness. These questions addressed in this paper are depicted in Figure 2 in PICO (P: Patient, I: Intervention, C: Comparison O: Outcome) format.

The PubMed, Embase, Web of Science, Google Scholar, and OVID databases were utilized to perform a literature review of appropriate studies published in the English language. The databases were queried using a search strategy including search term combinations of “breast implant illness,” “autoimmune,” and “systemic illness,” to investigate the relationship between autoimmune symptoms and breast implants. Article titles and abstracts were screened for relevance to breast implant illness due to unknown etiology, to exclude acute infection as causes. Due to the variety of labels given to breast implant illness, other search terms such as “ASIA” (referring to the label “autoimmune syndrome induced by adjuvants”), “breast implant adjuvant,” “breast implant,” and “connective tissue disease” were used to increase coverage of relevant articles. Retrospective and prospective cohort studies were included, with preference given to papers published within the past 10 years. Abstracts were excluded.

**Figure 3:**
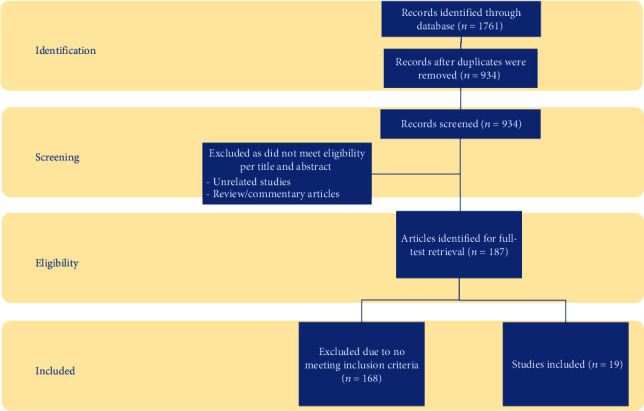
Summary of evidence search and selection.

The aforementioned databases were also interrogated for studies relevant to search terms “explantation” and “breast implant illness” to determine efficacy of explantation as a treatment for breast implant illness. Studies with patient samples of fifty or more were included. Case studies and review articles were excluded, but systematic reviews were included. The studies were scrutinized to ensure no overlap between the patient samples (Figure 3).

In the search relating to orthopedic devices and systemic symptoms, the databases were queried from January 1990 to July 2020 using a search strategy including the following search terms for literature on orthopedic implants: “orthopedic implant” AND “connective tissue disease,” “orthopedic implant” AND “systemic illness,” and “orthopedic implant” AND “ASIA.”

## 3. Breast Implant Illness and Associated Disorders

### 3.1. Symptoms Associated with Breast Implants

**Figure 4:**
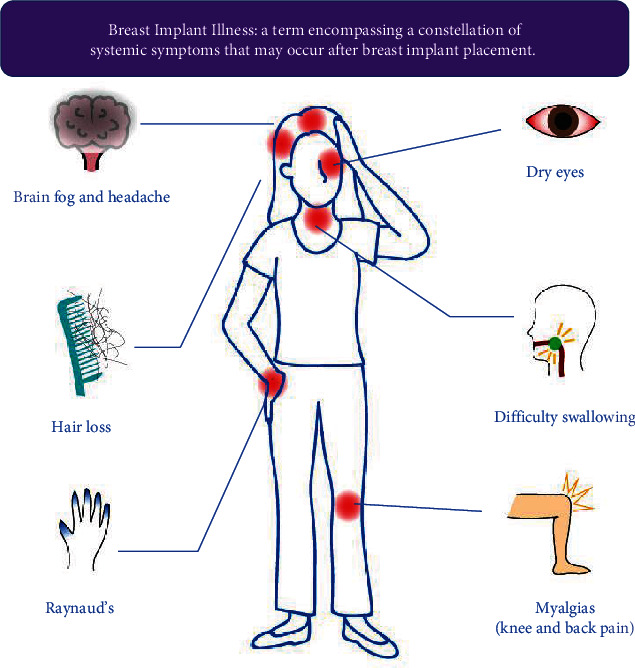
Depiction of commonly associated symptoms with breast implant illness.

Breast implant illness is an ill-defined label that has been adopted to refer to a variable constellation of symptoms after placement of breast implants. By nature, systemic illness is often hard to quantify, which is likely the reason for this lack of tangibility. Common symptoms reported in numerous papers include fatigue, arthralgia, muscle pain, memory loss, difficulty concentrating, rash, dry eyes, brain fog, and/or visual disturbance [36–39] (Figure 4). Other symptoms reported include dry mouth, difficulty swallowing, fever, facial flushing, paresthesia, and hair loss [1, 8, 37]. A study by Maijers et al. attempted to find patterns within the complaints described by women with breast implants but reported that the symptoms themselves seem to evade a common pattern [2]. Though a common pattern has been elusive, many of these symptoms have often been categorized as autoimmune-related and, in the following sections, may be reflected in symptoms correlated with specific autoimmune disorders.

There have been criticisms that assessment of symptoms associated with breast implants is unreliable due to the subjective bias in the self-reporting of these symptoms by women with breast implants [17]. While it may be true that it is difficult to measure objectively the increase in negative symptoms in breast implant recipients, it is clear that there is an overlapping pattern of these symptoms by thousands of individuals who have had implants [1, 8, 17].

### 3.2. Autoimmune Disorders Associated with Breast Implants

Parallel to the myriad of symptoms associated with breast implants, increased risk of developing autoimmune disorders has also been implicated with breast implants [15, 17, 20–23]. Watad et al. performed a large cross-sectional study investigating whether women with silicone breast implants were diagnosed with autoimmune or rheumatic disorders at higher rates [15]. They analyzed numbers of medical diagnoses in 24,652 silicone breast implant recipients compared with 98,604 age and socioeconomically matched women [15]. Women with silicone breast implants were conclusively associated with a higher likelihood of autoimmune or rheumatic disorders diagnosis, regardless of whether the breast implant was placed for reconstructive or cosmetic reasons. When comparing women with silicone breast implants with matched breast implant-free women, the hazard ratio of being diagnosed with at least one autoimmune/rheumatic disorder was 1.45 (95% CI 1.21-1.73), showing an increased risk of developing any autoimmune or rheumatic disorder in patients with breast implants [15].

Table 1 provides a summary of the large-scale research articles that have found a correlation between autoimmune disorders and breast implants. Below is a discussion of the autoimmune disorders that have most commonly been associated with breast implants by multiple studies in the past ten years.

### 3.3. Sjogren's Syndrome

Sjogren's syndrome is a chronic autoimmune inflammatory disorder in which the immune system targets the lacrimal and salivary glands in the body, among others [40]. It is commonly characterized by dry mouth and dry eyes due to diminished gland function, as well as dry pruritic skin and difficulty swallowing [40]. As noted in the discussion of symptoms associated with breast implants, symptoms similar to those found in Sjogren's syndrome are also commonly reported in women complaining of BII [15, 20, 21, 41].

Multiple epidemiological studies have found that women with breast implants are more likely to report subsequent diagnosis of Sjogren's syndrome. In a prospective cohort study by Contant et al., the percentage of women who reported symptoms related to Sjogren's syndrome increased from 11% preoperatively to 30% 1 one year after their breast implant surgery [41]. Another study reported an increased risk of Sjogren's syndrome in their comparative cohort study, with a risk ratio of 2.78 with adjustment for lifestyle factors [17]. In a 10-year retrospective cohort study with 55,279 patients with breast implants, authors reported an increased standardized incidence ratio (1.3), though not statistically significant, for Sjogren's disease [23]. A cross-sectional study by Watad et al. also reported an increased odds ratio of 1.58 for medical diagnosis of Sjogren's syndrome in breast implant recipients [15]. Balk et al. also conducted a systematic review of numerous epidemiological studies regarding breast implants and their association with medical illnesses. They reported an increased statistically significant risk ratio of 2.92 in their cumulative evaluation of seven studies investigating Sjogren's syndrome in association with breast implants [20]. Most recently, Coroneos et al. performed a retrospective cohort study in which they analyzed databases and looked at outcomes of 99,993 patients with breast implants. Breast implants were associated with increased rates of Sjogren's syndrome, with a standardized incidence ratio of 8.14 [21].

### 3.4. Rheumatoid Arthritis

Rheumatoid arthritis is an inflammatory polyarthritis that may lead to destruction of joints through erosion of cartilage and bone [40]. Its etiology may vary, but autoimmune dysfunction has been thought to be a factor. It is characterized by joint pain and stiffness. Rheumatoid arthritis was also more likely to be found in patients with breast implants [17, 20, 21, 36, 41]. In a prospective cohort study, it was reported that before the breast implant operation, 21% reported symptoms related to rheumatoid arthritis, while 40% reported those symptoms one year postoperatively, showing an increase in reports of these symptoms after breast implants were placed [41]. Balk et al. reported a statistically significant risk ratio of 1.38 in their systematic review of eleven studies investigating rheumatoid arthritis in patients with breast implants [20]. Another large-scale retrospective cohort study reported an increased risk for rheumatoid arthritis with breast implant recipients as well [21]. In a prospective cohort study by Lee et al., breast implants were associated with an 89% increase in risk for rheumatoid arthritis in an age-adjusted analysis, with the diagnoses confirmed with medical records [17]. The value did decrease to a 76% increase in risk after additional adjustment for BMI, smoking, and postmenopausal hormone use but still showed a positive risk in women with breast implants.

### 3.5. Systemic Sclerosis or Scleroderma

Systemic sclerosis is a chronic disorder that can manifest in multiple ways. Its hallmark feature is scleroderma, which is a thickened, hardened skin [40]. Almost all patients experience pain and fatigue and may also report capillary changes at the nail beds, skin ulcerations, edema, pruritus, and joint pain. While multiple areas of the body can be affected, it is generally the hands, fingers, and face that are involved [40].

Multiple recent studies have also found a correlation between breast implants and systemic sclerosis [15, 21, 22, 42]. They noted that the skewing in prevalence of these diagnoses suggested a causative role of breast implants in selectively triggering systemic sclerosis [22]. Watad et al. reported a significantly increased odds ratio of 1.63 for diagnosis of systemic sclerosis in their cross-sectional study [15]. In a retrospective cohort study, authors reported that they found systemic sclerosis was more common than rheumatoid arthritis in women with breast implants in their study cohort, even though the incidence of systemic sclerosis is lower than that of rheumatoid arthritis in the general population [22]. Another similar study also found an increased risk of scleroderma in women with breast implants, with a standardized incidence ratio of 7.0 [21]. Saigusa et al. noted in their investigation that the frequency of silicone breast implant history was significantly higher in a group of women with increased levels of anti-RNA polymerase III antibodies, which is a highly specific marker for systemic sclerosis [42], which led them to conclude there is an association between silicone breast implants and the development of systemic sclerosis.

### 3.6. Chronic Fatigue Syndrome

Chronic fatigue syndrome (CFS), also referred to as myalgic encephalomyelitis, is a condition with various presentations, the most prominent being easy fatigability [40]. Other symptoms include cognitive impairment, muscle aches, and sleep disruption. It has been noted that there is significant overlap between fibromyalgia and chronic fatigue syndrome, with about 70% of patients with fibromyalgia also qualifying for chronic fatigue syndrome, but it is still considered a separate entity at this time [22]. The etiology has not been definitively determined, but immune activation by subclinical viral or bacterial infection has been proposed as a possible contributing factor [22].

There have been a few studies that have linked breast implant placement to chronic fatigue syndrome [15, 22]. In the large meta-analysis by Watad et al., they found a significantly increased odds ratio of 1.37 [15]. However, in this study, they merged the number of diagnoses for both fibromyalgia and chronic fatigue syndrome to produce this value due to their overlap in symptoms. In their retrospective study, Khoo et al. compared the likelihood of new diagnosis of CFS in patients with breast implants with prior diagnosis of other autoimmune disorders such as SLE or systemic sclerosis [22]. They used patients with prior diagnoses of autoimmune disorder to rigorously control for the possibility that the patients were undiagnosed with autoimmune disorder prior to breast implant placement. Even with such meticulous consideration for confounding factors, they still found a 10% increase in diagnosis of chronic fatigue syndrome in patients with breast implants compared to a 2.22% increase in patients with SLE. One study failed to find an association between chronic fatigue syndrome and breast implants, but this was not statistically significant and therefore does not rule out the possibility of the association [23]. There is increased risk of lymphadenopathy in patients with breast implant illness [43, 44]. However, the studies discussing association of chronic fatigue syndrome with breast implant illness did not mention lymphadenopathy as a diagnostic factor for chronic fatigue syndrome.

### 3.7. Raynaud's Syndrome

Raynaud's syndrome is a disorder characterized by color changes of the skin of the digits, thought to be caused by abnormal vasoconstriction of digital arteries [40]. The exact etiology is not completely understood. Balk et al. reported a risk ratio of 1.33 in their systematic review of eleven studies looking at rates of Raynaud's syndrome in breast implant recipients, though they noted that it was not statistically significant [20]. Studies looking at reported symptoms in BII also reported an increased risk of Raynaud's syndrome in women with breast implants [1].

### 3.8. Autoimmune Serology

Increased risk for the presence of autoantibodies has been documented in the literature as well. Immunological serologies of women with breast implant illness symptoms diagnosed with these autoimmune and rheumatological disorders are limited in current literature, but there have been studies that show that there is increased risk of positive immunological factors in women with breast implant illness symptoms as well. These serologies include antinuclear antibodies [7, 45, 46], anticardiolipin [46], and anti-G-protein-coupled receptors [47], which have been associated with multiple autoimmune disorders, as well as antirheumatoid factor antibodies [7, 45, 46], which have been associated with rheumatoid arthritis. There is also increased risk of anti-RNA polymerase III, which is associated with systemic sclerosis [42, 46], and anti-dsDNA [45, 46], which is associated with systemic lupus erythematous. However, antibodies are often nonspecific to autoimmune disorders, and while their existence may support a diagnosis, their absence does not rule out the diagnoses of these disorders. This data can support that breast implants can trigger immunologic activation, so future research into immune serology in breast implant illness may be informative.

### 3.9. Are the Symptoms Breast Implant Specific or Manifested in Other Implants as Well?

Evidence has shown that the systemic symptoms associated with breast implants are not solely limited to silicone implants. There have been studies showing that these symptoms can be found in implants of a vast range of material. Multiple population-based studies included women with history of either silicone or saline implants in their study sample and reported no difference in the occurrence of BII between women with saline versus silicone implants [1, 23]. A case series by Alijotas-Reig et al. detailed 45 women with history of some type of biomaterial injection or implant [30]. These biomaterials included medical grade silicone, polyalkylimide, poly-L-lactic acid, and hyaluronic acid with or without methacrylate, which were placed in the breasts, face, buttocks, lower legs, and pectorals. The symptoms were largely consistent regardless of implant material, with arthralgias, general weakness, and myalgias as the most common complaints. Similarly, symptoms of breast implant illness were also seen in populations with material inserts of polyacrylamide hydrogel [48]. In many of these studies, they characterize the systemic symptoms as autoimmune/inflammatory syndrome induced by adjuvants (ASIA) [30, 48].

**Figure 5:**
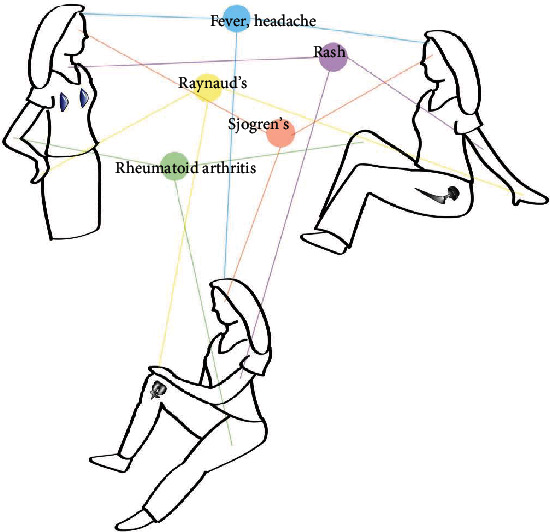
Depiction of overlap of symptoms between materials of different implants.

Expanding further, the systemic symptoms characterizing breast implant illness can also be found in some cases of orthopedic implants [33–35, 49]. A large-scale retrospective cohort study investigating long-term effects of artificial hip and knee joint implants was performed by Mellemkjaer et al., which looked at 24,636 patients with osteoarthritis who underwent hip implant surgery and 5,221 patients who received knee implants during 1977-1989, compared with rate in the general population in Denmark, and with that among osteoarthritis (OA) patients without implant surgery [34]. They found increased rates for Sjogren's syndrome (1.9 vs. 1.1 for Sjogren's syndrome for OA patients with hip surgeries) and systemic lupus erythematosus (SLE, 2.3 vs. 0.5 for SLE for OA patients with hip surgeries) for patients with hip implants. They also observed an increased risk of developing Sjogren's (2.4 vs 0.7 for Sjogren's for OA patients with knee surgeries) in patients with knee implants. Signorello et al. compared incidence of autoimmune or connective tissue disorders in their study of 101,771 hip implant recipients and 23,891 knee implant recipients with no previous known history of such disorders, with the general population [33]. They found an increased risk in both knee implants (standardized incidence ratio, SIR 1.7, 95% 1.5-1.9) and hip implants (SIR 1.1, 95% 1.1-1.2) for diagnosis of any autoimmune or connective tissue disorder after implant placement. A case control study by Laing et al. looked at 205 women diagnosed with undifferentiated connective tissue disorder (UCTD) and 2095 age and ethnicity-matched healthy controls, in order to determine which cohort had more exposure to either silicone or metal-containing implants [35]. Patients were considered to have UCTD if the review of their medial record identified signs and symptoms and/or lab abnormalities that suggested a systemic rheumatic disease but not sufficient to meet classification criteria for any connective tissue disease. Their results showed that a significant association was observed for patients with silicone-containing devices (odds ratio, OR 2.81), as well those with artificial joints (OR 5.01) and orthopedic metallic fixation devices (OR 1.95). This increase in incidence of UCTD in implant recipients persisted when controlled for numerous confounding factors. Etiology for this observed increased risk of autoimmune symptoms and immune sensitivity in orthopedic device implants has also been undetermined [49, 50] (Figure 5).

A wide array of implant materials all cause similar symptoms among them [20, 30, 33, 34]. This evidence contradicts directly implicating the implant material (e.g., silicone) as the cause. Subsequently, we are led to ask the question: is the autoimmune response independent of the implant material and in fact more dependent on the microenvironment around the implant?

## 4. Possible Causes

**Figure 6:**
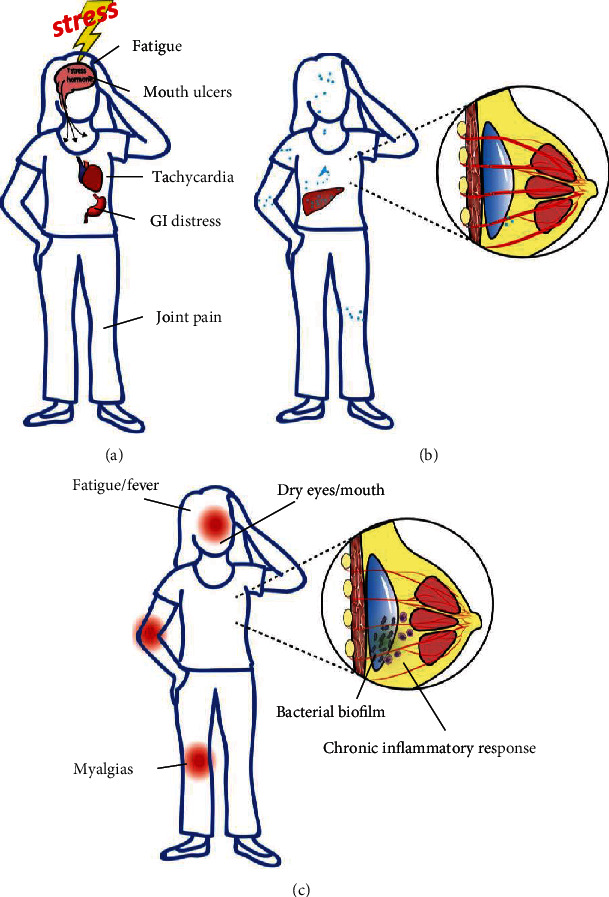
Depiction of proposed causes for breast implant illness. (a) Breast implant illness as a psychosomatic response to mental stress. (b) Breast implant illness as an immune reaction to silicone particulate leeching. (c) Breast implant illness as an activation of the immune system in response to biofilm formation on the breast implant surface.

The symptoms are primarily proposed to be caused by an autoimmune or immune response. The debate is over what could be causing this response—could it be the silicone particles of the implant itself, bacterial biofilm formation on and around the implant, or just a psychosomatic manifestation? [36, 51] The discussed hypotheses are illustrated in Figure 6.

### 4.1. Breast Implant Illness as a Psychosomatic Syndrome

Although patients receiving implants are diverse, the demographic of implant recipients has broadly been characterized by Ahern et al. and other research groups as more likely to have poor self-esteem and psychological problems [52]. This contributed to the hypothesis that breast implant illness symptoms may not be physiological but a result of increased anxiety and somatization. Systemic symptoms found in the autoimmune disorders associated with breast implant illness include fatigue, myalgias, and fever [20, 53]. These symptoms are also seen in stress-mediated somatization [52]. Given that medical implant procedures are generally considered invasive procedures, they often illicit increased stress and feelings of lack of control in patients undergoing these procedures. It has been argued that this mental burden may prompt the symptoms described in breast implant illness, as seen in Figure 6(a) [54]. Ahern et al. conducted a questionnaire, with results that showed that patients with breast implants had significantly higher anxiety than both healthy controls and medical/surgical inpatients [52]. They also noted breast implant patients scored higher for having anxious personality characteristics, showing that breast implant patients tend to worry more in general. Increased rates of mental illness have also been reported in women receiving cosmetic breast implants, as well as increased rates of admission due to psychiatric problems in women prior to breast implant surgery [55].

However, in the population studied by Ahern et al., 82% of the women had implants placed for cosmetic reasons, which they argue is the confounding factor for anxious personality characteristics. Yet multiple studies have shown that breast implant illness symptoms are found at rates that are not significantly different between women who had cosmetic or reconstructive reasons for breast implant surgery [20], which shows this hypothesis exhibits poor consistency across a more diverse patient cohort. Also, the data reported upon by Watad et al. has been compiled from medical records with clinically diagnosed autoimmune disorders by physicians, not solely patient-reported symptoms, which further detracts from the strength of this hypothesis. Another contradiction to this hypothesis is breast implant illness symptoms which have been recorded to arise many years after the surgery, with symptoms developing over 20 years after the operation in some patients [1]. Psychological stress triggered by surgery as a cause for BII would be more likely temporally be more directly following the operation. This evidence counters a psychosomatic etiology and legitimizes the symptoms found in breast implant illness experienced by thousands of women. This indicates that further investigation into actual physiological pathways is warranted before relegating breast implant illness as a somatic syndrome.

### 4.2. Is Implant Rupture and Silicone Leeching the Cause of Breast Implant Illness?

A prominent hypothesis in current literature purports that the symptoms of breast implant illness may be caused by leeching of silicone particles from the breast implants, as illustrated in Figure 6(b). There are reports that noted the presence of silicone particulates in the liver and spleen, as well as mediastinal, axillary, and internal mammary lymph nodes, after breast implant rupture [56]. Cohen Tervaert et al. hypothesized that a chronic inflammatory reaction can result from this migration of silicone gel bleed and lead to symptoms described in breast implant illness [12].

Other reports show that although silicone bleed is undeniable, it has been found that silicone is a biologically inert material, and it is therefore highly questionable whether silicone implants can lead to the widespread complaints of patients implanted with them [53, 57, 58]. Preliminary studies have also shown that there is little association of heightened cellular immune reactivity for silicone in women with silicone breast implants. In an article by Ellis et al., blood samples from 26 women with a history of silicone breast implants were tested for peripheral blood mononuclear cell reactivity against control, connective tissue proteins, and compounds in silicone implants and compared to measures for age-matched controls [57]. While the study found increased frequency and intensity of cellular immune responses against collagen I, collagen III, fibrinogen, and fibronectin, they found no significant difference in reactivity against the silicone antigens silicone dioxide, octamethylcyclotetrasiloxane (D4), and silicone gel [57]. The increased cellular immune response against collagen and fibrin products seems to support a cell-mediated connective tissue disorder. Further characterization of the cellular immune response is thus warranted in future studies.

A study of 90 women who were examined for an association between extracapsular silicone bleed and breast implant-associated illness also casts doubt on the hypothesis that silicone leeching is causing BII [58]. No statistically significant difference in complaints were found between a group of patients with magnetic resonance spectroscopy-confirmed evidence of silicone in the liver versus a group of patients without silicone detected in the liver. The study concluded that implant integrity has little impact on clinical symptoms [58]. A similar study analyzing adverse health outcomes according to breast implant rupture in 238 women found no difference in occurrence of self-reported diseases or symptoms between the women with ruptured implants and women with intact implants [53]. They also investigated serum levels of specific autoantibodies, such as antinuclear antibodies and rheumatoid factor, and found no statistically significant difference in levels of these antibodies in women with ruptured implants when compared to those with intact implants [53]. These studies suggest that silicone leeching throughout the body is unlikely the primary etiology to the development of the systemic symptoms after implant placement.

### 4.3. Implants and Bacterial Biofilm

Another hypothesis (illustrated in Figure 6(c)) is that bacterial biofilm formation on the implant leads to a host-pathogen interaction for a long period, which may trigger a chronic inflammation that leads to systemic autoimmune symptoms. Bacterial biofilms are sensitive to the host microenvironment in which they reside [59–61]. Bacterial biofilm has been connected already with a number of complications associated with implanted biomaterials [62–64]. Multiple studies have strongly associated bacterial biofilm with breast implant-associated anaplastic large cell lymphoma (BIA-ALCL) [65], as well as capsular contracture [62]. Orthopedic failure such as pedicle loosening and pain after years following implant placement has also been associated with bacterial biofilm [49, 63, 64]. Therefore, this hypothesis is compelling because bacterial biofilm has been implicated with multiple other implant complications.

Lee et al. investigated whether bacterial biofilm is associated with breast implant illness in their study of 50 women with BII symptoms [36]. They identified positive bacterial cultures from samples of the explanted breast implants and their capsules in a higher proportion of the cohort with breast implant illness than the control group. Cultures were obtained by grinding the capsule samples and their implant shells, followed by microbiological analysis. The most commonly identified organisms were *Cutibacterium acnes* and *Staphylococcus epidermidis. Cutibacterium acnes* is known to colonize lipid-rich areas of the body, often in the lipid-rich environment of the pilosebaceous glands of the skin [66]. Both *C. acnes* and *S. epidermidis* have been identified to produce a biofilm on orthopedic and cardiac prosthetics [67], and *S. epidermidis* has been shown to be a common cause of acute infection after breast implant surgery [68]. In a study by Katsnelson et al., cultures of breast implants were obtained during the explantation procedure through a capsulotomy made to access the implant pocket [39]. Positive culture results with BII symptoms were most commonly associated with the *Staphylococcus* genus [39]. In a recent report by Khan et al., authors noted that biofilm-forming bacteria *S. epidermidis* in breast implants results in host immune activation [69]. The formation of biofilm of both *S. epidermidis* and *C. acnes* has been previously established to be associated with a positive causative role in the pathogenesis of capsular contracture in breast implant patients as well [70]. With a scarcity of comprehensive research in this hypothesis, more research is necessary to determine the extent of the involvement of bacteria biofilm in breast implant illness.

## 5. Explantation as Treatment for Alleviating Symptoms Associated with Breast Implants

The systemic symptoms of breast implant illness are a major risk factor for eventual surgery for explantation of the breast implant, with a risk ratio of 5.6 in patients with symptoms including myalgias, arthralgias, fatigue, neuropathy, or cognitive impairment [71]. Currently, explantation has been shown to be effective in relieving symptoms in most patients with breast implant illness [5, 6]. de Boer et al. performed a systematic review in which data was extracted from 23 studies, 11 of which were case reports or case studies and 12 were cohort studies, investigating the effectiveness of implant removal as treatment for patients with complaints possibly related to their silicone breast implants [5]. Explantation of the silicone breast implant improved complaints in 75% of patients (469/622). In patients with autoimmune diseases, improvement was observed without additional immunosuppressive therapy in only 16% (3/18) of the patients. With the addition of immunosuppressive therapy, 56% of patients (10/18) improved. Other studies found similar results, with over 50% of patients experiencing relief of symptoms [2, 5, 7, 17, 39, 72, 73]. A summary of the studies investigating explantation efficacy is shown in Table 2.

### 5.1. Is Explantation a Proper Treatment?

However, explantation should not be considered the gold standard for treatment of breast implant illness. Many patients opt for breast implants for either cosmetic or reconstructive surgery. In the patient cohorts who choose to get breast implant placement after breast cancer treatment or prophylactic mastectomy, these implants may be important for their quality of life and body image. Evaluations of women after implant placement have shown that breast implants, particularly silicone breast implants, improve patient satisfaction in recipients [27–29]. It has also been documented that women who undergo explantation reported decreased appearance satisfaction levels and decreased confidence in their appearances [29, 74]. Removing the implant postmastectomy without replacing with a more invasive autologous reconstruction results in a chest deformity. Implant removal postcosmetic breast augmentation results in skin ptosis from stretching from the implant [29]. Addressing the skin ptosis requires a mastopexy leading in several additional extensive scars and cost. We hope that with further understanding of the etiology behind the link between implants and autoimmune disease, treatment regimens other than explantation can be conceived and developed. Although explantation with capsulectomy may help relieve the symptoms of these patients, a targeted approach addressing the etiology may make implant removal unnecessary.

## 6. Conclusion

Our review has found that autoimmune and systemic symptoms have been associated with breast implants in multiple large population studies in the past ten years. Similar autoimmune symptoms and diseases found in breast implant illness have also been associated with other implants such as orthopedic implants, as well as implants composed of other biomaterials. Currently the gold standard for treatment of breast implant illness is removal of the implant by explantation. However, this is not ideal because many women who get implants opt for them for enhancing quality of life and improved body image, both in cosmetic and reconstructive surgery after mastectomy. Simply removing the implant is a poor long-term treatment for BII and infeasible for orthopedic implants.

Limitations of this literature review include extracting information from existing studies with their own biases and limitations. We also chose to prioritize inclusion of research articles that were published in the past 10 years, but this may have led to exclusion of papers with relevant information as well. Therefore, these limitations should be kept in mind when interpreting the discussion of this paper.

This study has illuminated the need for further research into the evolving axis of surgical implant-mediated bacterial biofilm-induced host autoimmunity. Research in this domain will open possibility for rigorous protocols to decrease bacterial biofilm or modulate immune response which can be developed to address these symptoms without explantation.

## Figures and Tables

**Table 1:** Summary of included studies investigating association between breast implant placement and autoimmune/connective tissue disorders.

Author, year	Study type	Patients/studies	Findings
Balk et al. [20], Ann Intern Med., PMID: 26550776	Systematic review	32 studies	Increased risk of RA, Sjogren's syndrome, and Raynaud's in women with breast implants
Coroneos et al. [21], Ann Surg., PMID: 30222598	Retrospective cohort study	99,993 patients, (83317 silicone, 16676 saline)	Increased rates of Sjogren's syndrome, scleroderma, and RA in breast implant patients vs. general population
Khoo et al. [22],, Clin Rheumatol, PMID: 30706290	Retrospective cohort study	30 breast implants vs. 90 SLE or SSc controls	Significantly increased risk for fibromyalgia or CFS against SLE controls
Lee et al. [17], Int J Epidemiology, PMID: 20943932	Prospective cohort study	23847 patients (3950 with breast implants, 19897 controls)	Increased risk for CTDs confirmed with medical records with breast implants
Singh et al. [23], Plast Reconstr Surg, PMID: 28953716	Retrospective cohort study	55,279 breast implants	Increased risk for Sjogren's syndrome, RA, and mixed connective tissue disease, though not statistically significant
Watad et al. [15], Int J Epidemiology, PMID: 30329056	Retrospective cohort study	24652 SBI recipients vs. 98604 controls	Increased risk of having any autoimmune/rheumatic disorder with SBI, with significantly increased risk for Sjogren's syndrome, systemic sclerosis, and sarcoidosis

RA = rheumatoid arthritis; CFS = chronic fatigue syndrome; CTD = connective tissue disorder; SBI = silicone breast implant.

**Table 2:** Summary of studies showing improvement of breast implant illness symptoms with explantation.

Author, year	Study type	Patients/studies	Findings
Maijers et al. [2], Neth J Med, PMID: 24394743	Descriptive cohort study	*n* = 80 women with SBIs and subsequent onset of unexplained symptoms	After explantation, 36/52 (69%) women with systemic symptoms experienced significant reduction in symptoms
Colaris et al. [7], Immunol Res., PMID: 27406737	Comparative study	*n* = 100 patients diagnosed in 2014 with ASIA	54 patients underwent explantation, of which 27 (50%) experienced improvement of complaints
de Boer et al. [5], Immunol Res., PMID: 27412295	Systematic review	23 studies were analyzed, *N* = 622 patients who underwent explantation of breast implants	Explantation improved complaints in 469 of 622 (75%) patients. In patients with autoimmune diseases, additional immunosuppressive therapy increased improvement
Lee et al. [36], Plast Reconstr Surg Glob Open, PMID: 32440423	Prospective cohort study	*n* = 50 patients with BII	Explantation improved or resolved some symptoms in 39 out of 44 patients (78%)
Wee et al. [73], Ann Plast Surg, PMID 32530850	Retrospective cohort study	*n* = 552 women with SBIs undergoing explantation with post-operative symptom follow-up	Improvement across 11 common symptom domains following removal of breast implants and total capsulectomy
Glicksman et al. [72], Aesthetic Surg J, PMID: 34915566	Prospective cohort study	*n* = 150 women undergoing explantation of SBIs versus women undergoing mastopexy	Symptoms of BII significantly improved for greater than 6 months in women undergoing explantation of SBI
Katsnelson et al. [39], Plast Reconst Surg Glob Open, PMID 34513545	Retrospective cohort study	*n* = 248 women with SBIs undergoing explantation	Of 46 women who responded to postoperative symptom follow-up, 96% reported improvement in symptoms
